# Programmed death-1 mediates venous neointimal hyperplasia in humans and rats

**DOI:** 10.18632/aging.203185

**Published:** 2021-06-24

**Authors:** Peng Sun, Zhiwei Wang, Weizhen Liu, Mingxing Li, Shunbo Wei, Yanhua Xu, Zhentao Qiao, Wang Wang, Yang Fu, Hualong Bai, Jing’an Li

**Affiliations:** 1 Department of Vascular and Endovascular Surgery, First Affiliated Hospital of Zhengzhou University, Henan, China; 2 Department of Physiology, Medical School of Zhengzhou University, Henan, China; 3 Key Vascular Physiology and Applied Research Laboratory of Zhengzhou City, Henan, China; 4 School of Material Science and Engineering & Henan Key Laboratory of Advanced Magnesium Alloy & Key Laboratory of Materials Processing and Mold Technology, Ministry of Education, Zhengzhou University, Henan, China; 5 Department of Internal Medicine, First Affiliated Hospital of Zhengzhou University, Henan, China; 6 Department of Gastrointestinal Surgery, First Affiliated Hospital of Zhengzhou University, Henan, China

**Keywords:** PD-1, lymphocyte, proliferation, neointimal hyperplasia, patch venoplasty

## Abstract

Venous neointimal hyperplasia can be a problem after vein interventions. We hypothesized that inhibiting programmed death-1 (PD-1) can decrease venous neointimal hyperplasia in a rat inferior vena cava (IVC) patch venoplasty model. The rats were divided into four groups: the control group was only decellularized without other special treatment; the PD-1 group was injected with a single dose of humanized PD-1 antibody (4 mg/kg); the PD-1 antibody coated patches group; the BMS-1 (a PD-1 small molecular inhibitor) coated patches group (PD-1 inhibitor-1). Patches were implanted to the rat IVC and harvested on day 14 and analyzed. Immunohistochemical analysis showed PD-1-positive cells in the neointima in the human samples. There was high protein expression of PD-1 in the neointima in the rat IVC venoplasty model. PD-1 antibody injection can significantly decrease neointimal thickness (*p* < 0.0001). PD-1 antibody or BMS-1 was successfully conjugated to the decellularized rat thoracic artery patch by hyaluronic acid with altered morphology and reduced the water contact angle (WCA). Patches coated with humanized PD-1 antibody or BMS-1 both can also decrease neointimal hyperplasia and inflammatory cells infiltration. PD-1-positive cells are present in venous neointima in both human and rat samples. Inhibition of the PD-1 pathway may be a promising therapeutic strategy to inhibit venous neointimal hyperplasia.

## INTRODUCTION

After vascular interventions, neointimal hyperplasia occurs and can cause treatment failure; scientists and surgeons have attempted to identify an effective pharmacological method to suppress neointimal hyperplasia [1, 2]. Among the different cell types in the neointima after intervention, mature vascular smooth muscle cells are the major cellular source of intimal hyperplasia in vein grafts [3]. Therefore, paclitaxel- or rapamycin-coated balloons and stents have been widely used to decrease neointimal hyperplasia by inhibiting neointimal smooth muscle cell proliferation [4, 5]. Both drugs have been used for more than twenty years and have contributed to improved patency rates after artery interventions, although there have been some disagreements [5]. Vascular patches are used during surgery to repair or reconstruct damaged blood vessels [6]. Venous patch angioplasty is often used in the conditions when the inferior vena cava (IVC) or hepatic veins have been injured [7, 8]. However, intimal hyperplasia of the venous patch is also an important cause of graft failure [9].

Humanized antibodies to treat human cancers and other diseases are a milestone in modern therapeutic strategies, and programmed death-1 (PD-1) is a representative of these novel antibodies [10]. We previously showed that a PD-1 antibody and BMS-1 could effectively decrease neointimal hyperplasia in a rat aortic patch angioplasty model and that PD-1 neutralization decreases macrophage and lymphocyte numbers in the neointima and decreases TGF β1 expression [11]. These findings provided a new strategy to inhibit neointimal hyperplasia after arterial intervention. Arteries and veins are two different systems, and there are high occlusion and failure rates after prosthetic grafts are implanted in the venous system [12]. In humans, we showed a substantially thicker neointima in the venous system than in the arterial system [13]. Venous bypasses also have low patency rates, with 5-year secondary patency rates of 86% for femoroiliac and iliocaval bypasses and 57% for femorocaval bypasses [14]. In rats, we showed a thicker neointima in the venous patch angioplasty model compared with the arterial patch angioplasty model [11], and rapamycin covalent pericardial patches inhibited venous neointimal hyperplasia after inferior vena cava (IVC) patch angioplasty in rats [9].

Based on this prior knowledge, we hypothesized that the inhibition of PD-1 could also decrease venous neointimal hyperplasia. We used a decellularized rat thoracic artery patch and a rat venoplasty model to test our hypothesis [9].

## RESULTS

**Figure 1:**
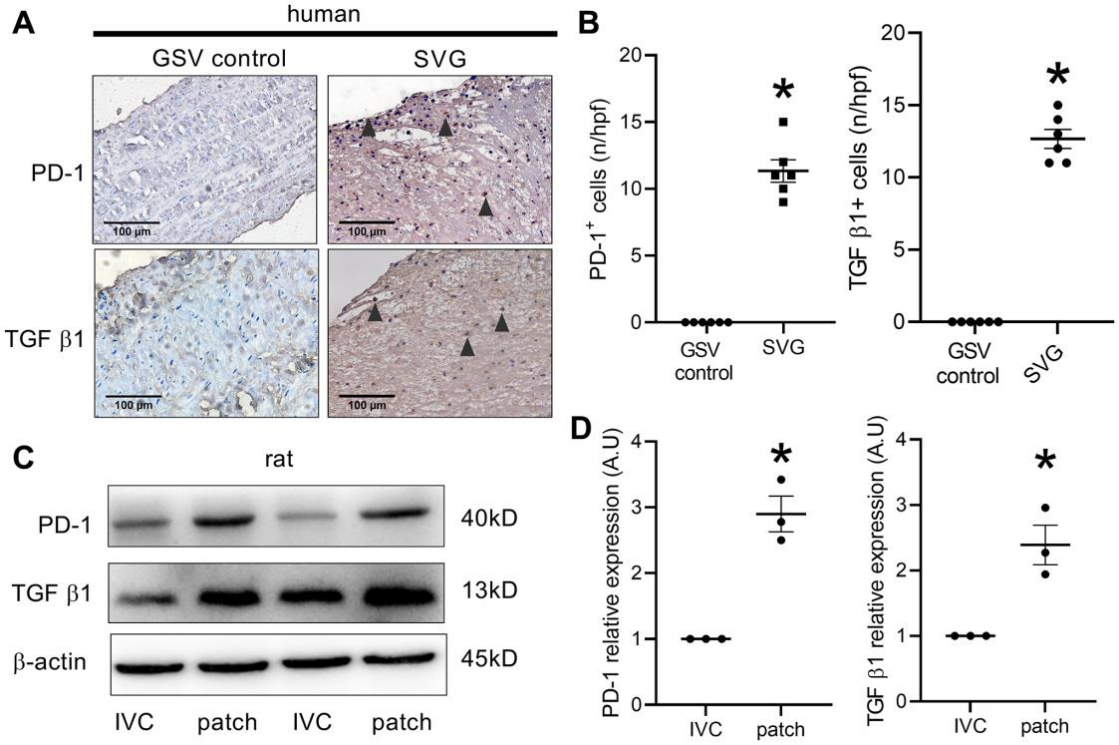
**PD-1 expression in human and rat venous neointimas.** (**A**) Immunohistochemistry images showing PD-1 and TGF β1 expression in the native human great saphenous vein (GSV control) and the neointima of spiral great saphenous vein graft (SVG); black arrowhead showing positive cells; scale bar, 100 μm; *n* = 3. (**B**) Bar graphs showing PD-1- (^*^p < 0.0001, *t*-test) and TGF β1-positive cells (^*^p < 0.0001, *t*-test) per high-power field in the human GSV and SVG neointima, *n* = 6. (**C**) Western blot showing the expression of PD-1, TGF β1, and β-actin in the rat IVC and the patch after patch venoplasty at day 14; *n* = 3. (**D**) Bar graph showing PD-1 (^*^p = 0.0022, *t*-test) and TGF β1 (^*^p = 0.0128, *t*-test) density; *n* = 3.

To explore whether there were PD-1 positive or TGF β1 positive cells in the human venous neointima, human spiral vein graft sections were sectioned and stained, there were neither PD-1-positive cells nor TGF β1-positive cells in the fresh human great saphenous vein; however, there were PD-1- and TGF β1-positive cells in the neointima of the human spiral vein graft sample (Figure 1A, 1B). We then examined PD-1 and TGF β1 protein expression in the rat IVC and the neointima of IVC venoplasty harvested on day 14. Western blot analysis showed increased PD-1 and TGF β1 protein expression in the neointima after patch angioplasty in rats (Figure 1C, 1D).

**Figure 2:**
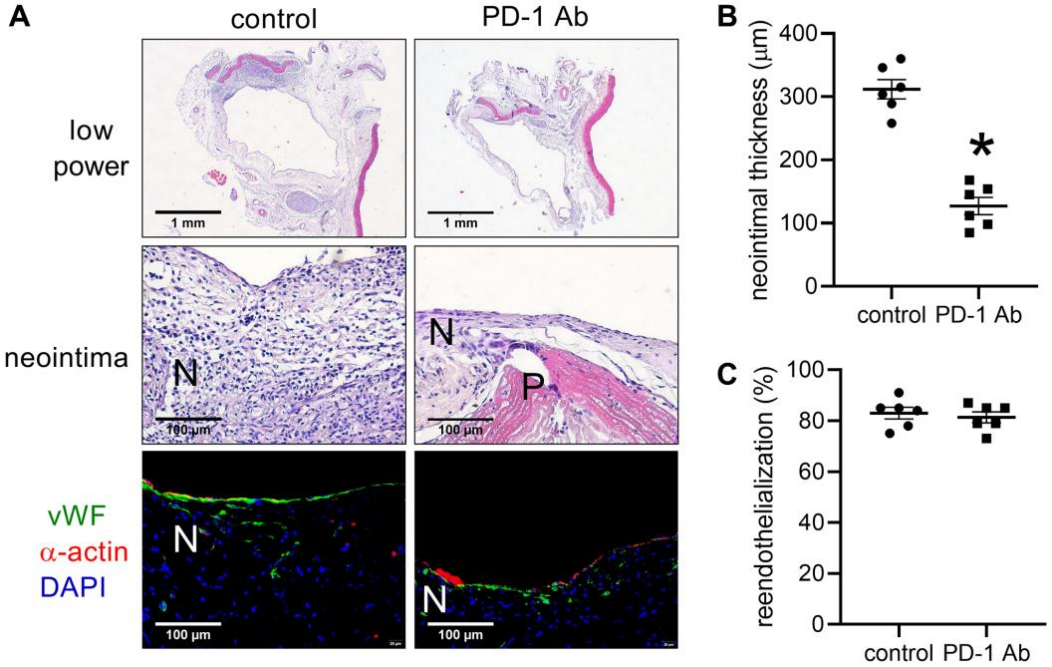
**Intraperitoneal (IP) injection of PD-1 decreases neointimal thickness after patch venoplasty in rats.** (**A**) Representative image of the patch stained with H&E at day 14; first row: a low-power image of H&E staining; second row: a high-power image of H&E staining showing the neointima; third row: merged immunofluorescence image of vWF (green) and α-actin (red) and DAPI (blue) staining showing the neointima. PD-1 Ab (IP injection of humanized PD-1 antibody group); P, patch; N, neointima; *n* = 6. (**B**) Bar graph showing neointimal thickness; ^*^p < 0.0001, *t*-test; *n* = 6. (**C**) Bar graph showing neointimal reendothelialization; *p* = 0.6104, *t*-test; *n* = 6.

Since the PD-1 antibody neutralization can decrease arterial neointimal hyperplasia [11], we next examined whether it could decrease venous neointimal hyperplasia. There was a thick neointima in the control group, but there was a significantly thinner neointima in the patches treated with PD-1 injection group (Figure 2A, 2B). There were both vWF- and α-actin-positive cells in the neointima, and there was no difference in the neointimal reendothelialization rate between the control and PD-1 antibody injection groups (Figure 2A, 2C).

**Figure 3:**
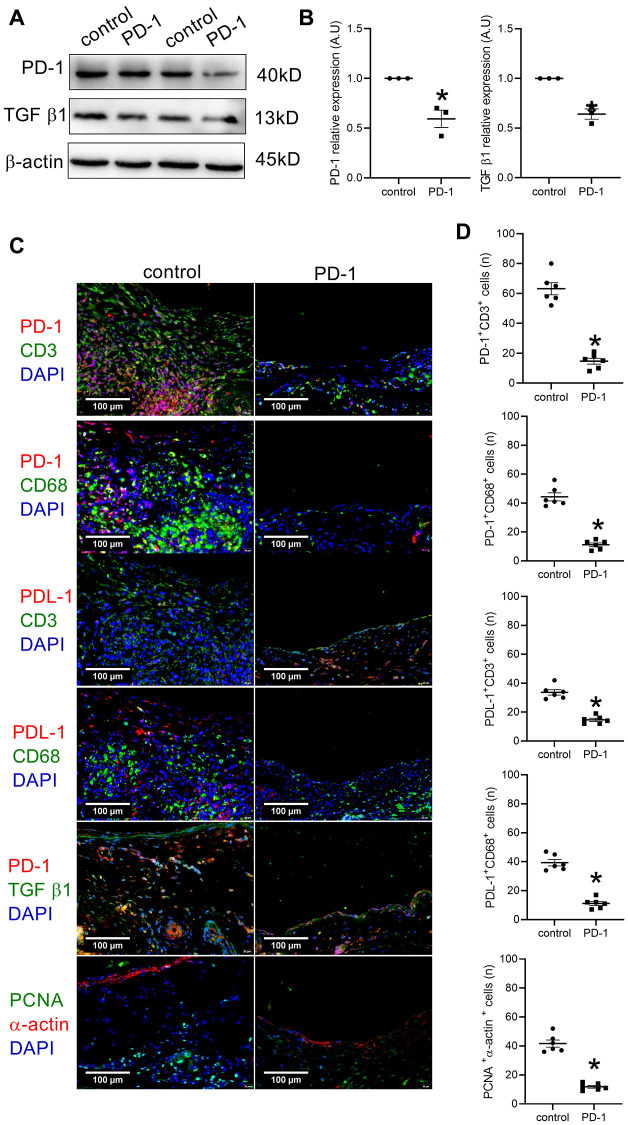
**Intraperitoneal (IP) injection of PD-1 antibody decreases PD-1 expression after patch venoplasty in rats.** (**A**) Western blot showing the expression of PD-1 and TGF β1 after the intraperitoneal injection of the PD-1 antibody in rat IVCs and neointimas at day 14; *n* = 3. (**B**) Bar graph showing PD-1 (^*^p = 0.0096, *t*-test) and TGF β1 (^*^p = 0.0023, *t*-test) density; *n* = 3. (**C**) Merged immunofluorescence images showing CD3 (green), PD-1 (red) and DAPI (blue); CD68 (green), PD-1 (red) and DAPI (blue); CD3 (green), PDL-1 (red) and DAPI (blue); CD68 (green), PDL-1 (red) and DAPI (blue); TGF β1 (green), PD-1 (red) and DAPI (blue); PCNA (green), α-actin (red) and DAPI (blue); scale bar, 100 μm; *n* = 6. (**D**) Bar graphs showing CD3 and PD-1 dual-positive cells (^*^p < 0.0001, *t*-test); CD68 and PD-1 dual-positive cells (^*^p < 0.0001, *t*-test), CD3 and PDL-1 dual-positive cells (^*^p <0.0001, *t*-test); CD68 and PDL-1 dual-positive cells (^*^p < 0.0001, *t*-test), PCNA and α-actin dual-positive cells (^*^p < 0.0001, *t*-test); *n* = 6.

We further examined whether PD-1 antibody injection could decrease PD-1 protein expression after patch angioplasty. Western blot analysis showed decreased PD-1 and TGF β1 protein expression after PD-1 antibody injection at day 14 (Figure 3A, 3B). There were significantly fewer PD-1 and CD3 dual-positive cells, and significantly fewer PD-1 and CD68 dual-positive cells in the neointima of the PD-1 antibody injection group than the control group (Figure 3C, 3D). PDL-1 is a ligand of PD-1, PD-1 affects immune cells by binding to PDL-1 in tumor cells. There were significantly fewer PD-L1 and CD3 dual-positive cells and fewer PD-L1- and CD68-positive cells in the neointima of the PD-1 antibody injection group than the control group (Figure 3C, 3D). PD-1 and TGF β1 expression, and α-actin and PCNA dual-positive cells were significantly decreased in the PD-1 antibody injection group (Figure 3C, 3D).

**Figure 4:**
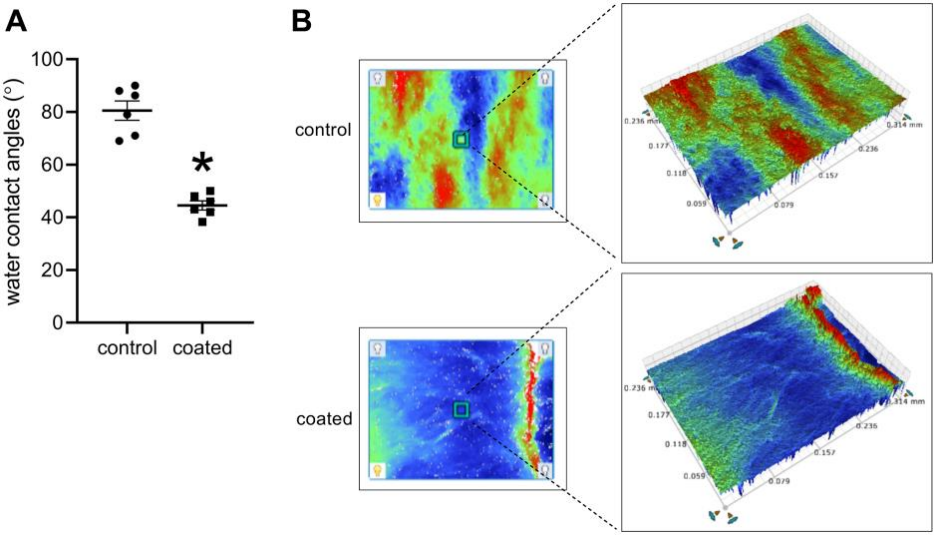
(**A**) Water contact angles, ^*^p < 0.0001, mean ± SD, *n* = 6. (**B**) 3D optical microscopy images of each sample, *n* = 6.

We then coated PD-1 antibody and BMS-1 onto the surface of decellularized rat thoracic patches and implanted them into the rat IVC. The coated patch surface showed a significantly smaller WCA than the control patch (Figure 4A). The 3D optical microscopy images showed that the coated patch and uncoated control patch displayed distinct surface morphologies, wherein the uncoated control patch showed complex surface morphology and different roughness distributions, which could be directly observed on the image by diversified colors. In contrast, the coated patch showed uniform morphology and roughness distribution, and almost the entire surface was covered in blue in the 3D optical microscopy image (Figure 4B).

**Figure 5:**
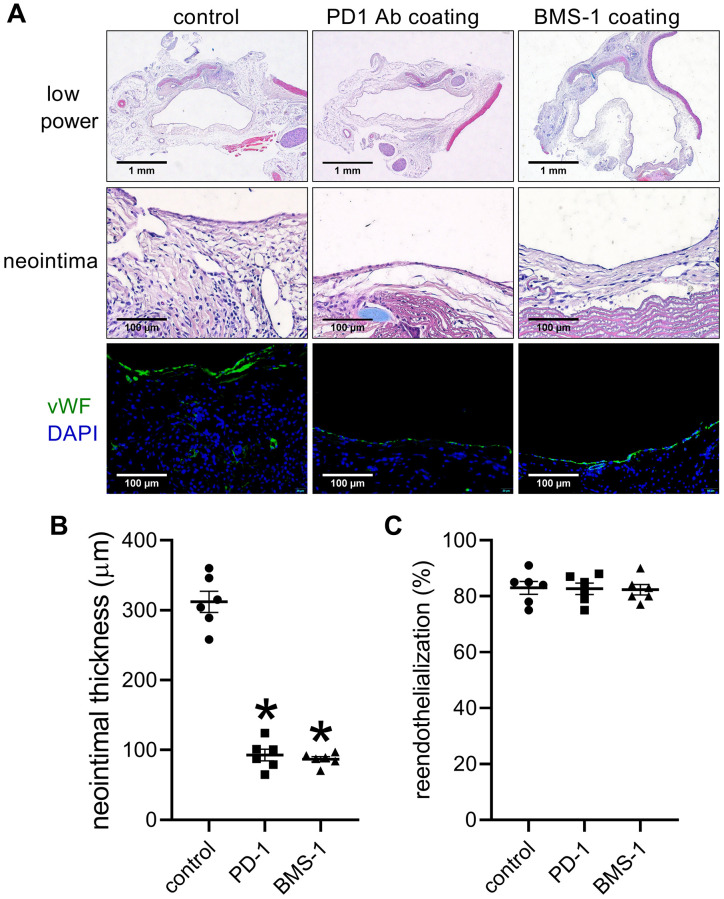
**PD-1- and BMS-1-coated patches decrease neointimal thickness after patch venoplasty in rats.** (**A**) Representative image of the patch stained with H&E at day 14; first row: a low-power image of H&E staining; second row: a high-power image of H&E staining showing the neointima; third row: merged immunofluorescence image of vWF (green) and DAPI (blue) staining showing the neointima; scale bar, 100 μm; *n* = 6. (**B**) Bar graph showing neointimal thickness; *p* < 0.0001, one-way ANOVA; ^*^p < 0.0001, Tukey's multiple comparisons test. *n* = 6. (**C**) Bar graph showing neointimal reendothelialization; *p* = 0.9746, *n* = 6. one-way ANOVA.

**Figure 6:**
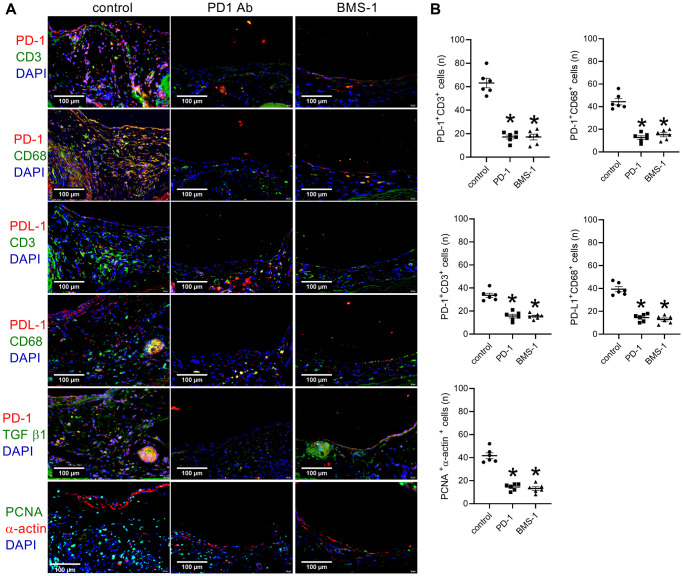
**PD-1- and BMS-1-coated patches decrease PD-1 expression after patch venoplasty in rats.** (**A**) Merged immunofluorescence images showing CD3 (green), PD-1 (red) and DAPI (blue); CD68 (green), PD-1 (red) and DAPI (blue); CD3 (green), PDL-1 (red) and DAPI (blue); CD68 (green), PDL-1 (red) and DAPI (blue); TGF β1 (green), PD-1 (red) and DAPI (blue); PCNA (green), α-actin (red) and DAPI (blue); scale bar, 100 μm; *n* = 6. (**B**) Bar graphs showing CD3 and PD-1 dual-positive cells (*p* < 0.0001, one-way ANOVA; ^*^p < 0.0001), CD68 and PD-1 dual-positive cells (*p* < 0.0001, one-way ANOVA; ^*^p < 0.0001), CD3 and PDL-1 dual-positive cells (*p* < 0.0001, one-way ANOVA; ^*^p < 0.0001), CD68 and PDL-1 dual-positive cells (*p* < 0.0001, one-way ANOVA; ^*^p = < 0.0001), PCNA and α-actin dual-positive cells (*p* < 0.0001, one-way ANOVA; ^*^p < 0.0001); *n* = 6.

After 14 days, there were substantially thinner neointimas in the PD-1- and BMS-1-coated patches than the control patches (Figure 5A, 5B). There were similar neointimal reendothelialization rates in these three groups (Figure 5A, 5C). Significantly fewer PD-1 and CD3 dual-positive cells and significantly fewer PD-1- and CD68-positive cells were in the coated patches compared to the control patches (Figure 6A, 6B); fewer PDL-1 and CD3 dual-positive cells and fewer PDL-1- and CD68-positive cells were found in the neointima of the coated patches compared to the control group (Figure 6A, 6B). α-actin and PCNA dual-positive cells were also significantly decreased in the coated groups (Figure 6A, 6B).

## DISCUSSION

In this study, we showed that PD-1 was expressed in the venous neointima in both humans and rats. Blocking PD-1 can also decrease macrophages and lymphocytes in the neointima and reduced neointimal thickness in a rat patch venoplasty model, this is like our previous research [11]. Compared to the commonly used drug rapamycin [9], these data suggest that PD-1 may be a novel treatment target after vein interventions. There is novelty in this research compared to our previous research [11], we previously showed there were difference of the neointima between arterial patch angioplasty and venous patch venoplasty in rat, there was a thicker venous neointima compared to the arterial neointima; the endothelial cells expressed venous markers in the venous system and expressed arterial markers in the arterial system [13, 15, 16]. There was also a difference between arterial neointima and venous neointima in human [17], since there are also a large number of venous procedures in clinic, so it is meaningful to find new method to decrease venous neointimal hyperplasia.

Different cells migrate and accumulate after vascular interventions, thus making the process of neointimal hyperplasia complex; current therapeutic protocols include antiplatelet and antithrombotic therapies [18, 19]. Devices such as rapamycin- and paclitaxel-coated balloons and stents have shown promising results [20, 21], suggesting that smooth muscle proliferation largely contributes to neointimal hyperplasia [3, 22]. Although these methods showed increased patency rates, none showed long-term success [23, 24]. Neointimal hyperplasia not only involves smooth muscle cells but also includes macrophage and lymphocyte accumulation [25, 26]. Previously, we showed that lymphocytes and macrophages accumulated in the human venous neointima [17]. In this study, we also showed a large number of CD3- and CD68-positive cells in the neointima in rats, but in the patches in the PD-1-treated or BMS-1-treated groups, there were very few CD3- and CD68-positive cells (Figure 3, Figure 6). Similar to the arterial neointima, CD3- and CD68-positive cells may also contribute to venous neointimal thickening [17]. TGF-β1 plays an important role in neointimal hyperplasia [27] and is expressed in rat aortic patch angioplasty; most of the PD-1-positive cells are TGF β1-positive cells, and CD3- and CD68-positive cells also express PD-1 [11]. In this rat venoplasty model, we showed that TGF β-positive cells were present in the venous neointima in both humans and rats, and CD3- and CD68-positive cells also expressed PD-1.

We used HA to modify the surface of the decellularized thoracic artery patch, and this method was effective in delivering molecules and drugs [28]. A smaller WCA leads to a larger contact area between material surfaces and blood, and more nutrients, such as protein, may be adsorbed onto the material surfaces, which is conducive to the adhesion, spreading, and proliferation of tissue repair-related cells [29]. Therefore, coated substrates are predicted to have improved effects on the tissue repair of lesions. In the coated patch, the 3D optical microscopy images showed uniform morphology and roughness distribution. Based on the significant changes in surface wettability and morphology, it can be concluded that the HA/BMS-1 coating was successfully prepared on the substrate [29].

There are some limitations in this research. First, this research only showed the neointima of rat IVC patch venoplasty model; other models like commonly used vein graft, prosthetic graft, balloon injury or stent implantation models need to be explored. Second, the animal used in this research is rat, other animals like mouse or other bigger animals should also be tested; since the diameter and velocity of the vessel can influence the neointimal hyperplasia. Third, we only showed the result of two weeks, longer time of observation is needed to see if there were any side effects of PD-1 treatment. Fourth, further experiments on interposition model should be tested.

In summary, PD-1-positive cells are present in venous neointima in both humans and rats, and the inhibition of PD-1 can decrease venous neointimal thickening. These data suggest that the inhibition of the PD-1 pathway may be a novel therapeutic strategy after vein interventions.

## MATERIALS AND METHODS

### Human samples

Human samples were obtained as described previously [17]. Briefly, a trauma patient required popliteal artery and vein reconstruction with a spiral saphenous vein graft (SVG) in the popliteal vein, amputation was performed on day 18 after vascular reconstruction because of the patient’s serious injuries, and all protocols involving human biospecimens complied with all relevant ethical regulations. Tissues were processed and stained as described previously, briefly, the SVG was fixed and embedded in paraffin and sectioned (4 μm thickness); sections were heated in citric acid buffer (pH 6.0) for antigen retrieval and then treated with 0.3% hydrogen for 30 min, then the sections were incubated with PD-1 or TGF β1 antibody; after overnight incubation, the sections were incubated with appropriate secondary antibodies for 1 hr at room temperature and treated with DAB Horseradish Peroxidase Color Development Kit (Beyotime, Shanghai, China) to detect the reaction products; finally, the sections were counterstained with hematoxylin (BASO) [17].

### Coating with hyaluronic acid and humanized PD-1 antibody or BMS-1

All experiments were approved by the Institutional Animal Care and Use Committee at Zhengzhou University and performed in accordance with the NIH guidelines for the care and use of laboratory animals (NIH Publication #85-23 Rev. 1985). Decellularized and coating procedures were carried out as we previously described [11]. Briefly, the thoracic aorta (TA) was harvested and incubated in 10 mL of sodium dodecyl sulfate buffer (1.8 mM sodium dodecyl sulfate, 1 M NaCl, and 25 mM EDTA in PBS) for 24 hours, and then rinsed with PBS. The coating procedure was also performed as previously described [11]. Briefly, the decellularized TA was immersed in hyaluronic acid (HA) solution (2 mg/ml. Bloomage Biotech, China) with a molecular weight of 100,000 Da. for 15 min. The HA solution was activated in advance in a water-soluble carbodiimide solution and incubated for 6 h at 37°C, and then the HA-coated samples were immersed in humanized PD-1 antibody (4 mg/ml, SHR-1210, Hengrui Medicine, Jiangsu, China) preactivated in water-soluble carbodiimide solution. HA/PD-1 were successfully coated onto the samples. BMS-1 (1 mg/ml, HY-19991, Med Chem Express) was coated in a similar fashion [11]. The wettability change in each sample surface was measured by determining the WCA (DSA 100, Krüss, GmbH, Germany) [30]. The morphology and roughness of each sample were observed by 3D optical microscopy (NPFLEX, Bruker, Madison, WI, USA) [31], and the different colors distributed on the surfaces represent different roughness levels and morphologies.

### Rat IVC patch venoplasty model

A rat IVC venoplasty model was used as previously described [9, 13]. Briefly, the rat IVC was exposed, and a 3-mm venotomy was made, the control patch and coated patch (4 mm × 2 mm) were sewn to the IVC using running 11-0 nylon sutures; the clamps were removed and the abdomen was then closed. The rats were injected with humanized PD-1 antibody (4 mg/kg; 4 mg/100 μL) in the PD-1 injection group. The patches were harvested for analysis on day 14. Neointimal and adventitial thickness were the mean of measurements from the surface edge to the edge of the patch in three independent areas.

### Tissue analysis

The rats were anesthetized, and tissues were fixed by transcardial perfusion of PBS followed by 10% formalin. The samples were fixed and then embedded in paraffin and sectioned (4-μm thickness). The tissue sections were deparaffinized and stained with hematoxylin and eosin (H&E; Baso, Zhuhai, China) according to the manufacturer’s recommendations.

### Immunohistochemistry (IHC) and immunofluorescence (IF) analysis

The sections were heated in a citric acid buffer (pH 6.0, Beyotime, Shanghai, China) at 100°C for 10 min for antigen retrieval. The sections were then treated with 0.3% hydrogen peroxide for 30 min in the IHC staining. The sections were then incubated overnight at 4°C with primary antibodies (Table 1). In the IHC staining, the sections were incubated with appropriate secondary antibodies (Table 1) for 1 hour at room temperature and then treated with a 3,3N-diaminobenzidine tetrahydrochloride (DAB) horseradish peroxidase color development kit (Beyotime, Shanghai, China) to detect the reaction products. Finally, the sections were counterstained with hematoxylin (Baso, Zhuhai, China). In the IF staining, the sections were incubated overnight at 4°C with primary antibodies (Table 1) diluted in dilution buffer (Beyotime, Shanghai, China). The sections were incubated with secondary antibodies (Table 1) for 1 hour at room temperature, after which the sections were stained with the fluorescent dye 4,6-diamidino-2-phenylindole (DAPI, Solarbio, Beijing, China) to mark cellular nuclei. Positive cell numbers were counted and blindly reviewed by three professional pathologists. Reendothelialization was determined as the length of CD31-positive cells divided by the length of the neointima. Positive cells were directly counted in 3 high-power fields in each sample, and the mean numbers of cells were then compared.

**Table 1:** Antibodies used in this experiment.

**Antibody**	**Vendor**	**Lot number**	**Concentration**
Primary antibody			
α-actin	abcam	Ab5694	IF:1:200
β-actin	ABclonal	AC026	WB:1:1000
CD3	Santa Cruz	SC-20047	IF:1:50
CD68	abcam	Ab31360	IF:1:100
PCNA	abcam	Ab29	IF:1:100
PD-1	ABclonal	A11973	IF,IHC:1:50 WB:1:500
PD-L1	ABclonal	A11273	IF:1:50
TGF β1	Santa Cruz	SC-130348	WB:1:100 IF:1:50
Secondary antibody			
Goat anti rabbit	bioworld	BS12478	1:100
Goat anti mouse	bioworld	BS13278	1:100
488 Goat anti mouse	ABclonal	AS073	1:200
CY3 Goat anti rabbit	ABclonal	AS007	1:200
488 Donkey anti rabbit	ABclonal	AS035	1:200
Rhodamine Donkey anti goat	ABclonal	AS069	1:200
488 Goat anti rabbit	ABclonal	AS073	1:200

### Western blotting

The patches were carefully harvested and snap-frozen in liquid nitrogen as we previously described [9, 16]. The samples were crushed and mixed with buffer containing protease inhibitors (Roche, Complete Mini 12108700) before sonication (5 sec) and centrifugation (135,000 rpm, 15 min). Equal amounts of protein from each experimental group were loaded for SDS-PAGE, followed by incubation with primary antibodies (Table 1) and secondary antibodies (Table 1), and the signals were detected using the electrochemiluminescence (ECL) detection reagent. The density of the blots was measured by Image J software (NIH).

### Statistical analysis

The data are expressed as the mean ± SEM. Statistical significance was determined by ANOVA and *t*-tests. *P*-values less than 0.05 were considered significant. The data were analyzed using Prism 6.0 software (GraphPad Software; La Jolla, CA, USA).

### Ethical approval

All applicable international, national, and/or institutional guidelines for the care and use of animals were followed.

### Consent for publication

Not applicable. All authors agree to publication, and there are no permissions needed.
